# Rhythm and Rest: Sex, Circadian Preference and Sleep During Aging

**DOI:** 10.1093/geroni/igaf122.3102

**Published:** 2025-12-31

**Authors:** Natasa Billeci, Ashley Curtis

**Affiliations:** University of South Florida, Tampa, Florida, United States; University of South Florida, Tampa, Florida, United States

## Abstract

Circadian rhythms and sleep health are linked to cognitive decline and Alzheimer’s disease (AD) risk. Sex differences exist in sleep and circadian patterns, with men experiencing greater declines in non-rapid eye movement (NREM) sleep and women reporting more sleep disturbances and insomnia. However, the interactive role of sex in circadian preference and polysomnography (PSG)-assessed sleep remains unclear. Understanding these relationships is critical, as women constitute 67% of AD cases. This study examined sex differences in the association between circadian preference and objective sleep parameters in older adults. Cognitively healthy older adults (N = 66; 38 women; Mage=68.05, SD = 5.70) completed overnight PSG and the Morningness-Eveningness Questionnaire. Moderated regressions tested interactions between sex and circadian preference on PSG-measured sleep outcomes, including sleep onset latency, wake time after sleep onset, total sleep time, %N1-N3, and %REM, controlling for age, anxiety, depression, and apnea-hypopnea index. Sex significantly moderated the association between circadian preference and %N3 sleep (R²=0.08, p=.01). In women, greater eveningness preference was associated with lower %N3 (B = 0.80, SE = 0.39, p=.047), while no such association was observed in men (p=.10). The findings suggest that circadian preference and slow-wave sleep relationships may be sex-dependent. Women with an eveningness preference may have a lower %N3, a pattern linked to increased AD risk. Monitoring slow-wave sleep in these individuals could aid in early intervention. Further research should explore sex-specific mechanisms, including postmenopausal hormone changes, circadian gene polymorphisms, and metabolic influences on aging sleep patterns.

